# Supplementary material for: Somatic DNA Variants in Epilepsy Surgery Brain Samples from Patients with Lesional Epilepsy
Source: Int J Mol Sci. 2025 Jan 19;26(2):815. doi: 10.3390/ijms26020815 (PMC11766355; doi:10.3390/ijms26020815)
Supplement: Supplementary file 1 [file ijms-26-00815-s001.zip › 20241216_Supplementary_figures.pdf]

SUPPLEMENTARY FIGURES to article

Somatic DNA Variants in Epilepsy Surgery Brain Samples from Patients with Lesional Epilepsy

Jana Marie Schwarz, Lena-Luise Becker, Monika Wahle, Jessica Faßbender, Ulrich Thomale, Anna Tietze, Susanne Morales-Gonzalez, Ellen Knierim, Markus Schuelke, Angela M. Kaindl

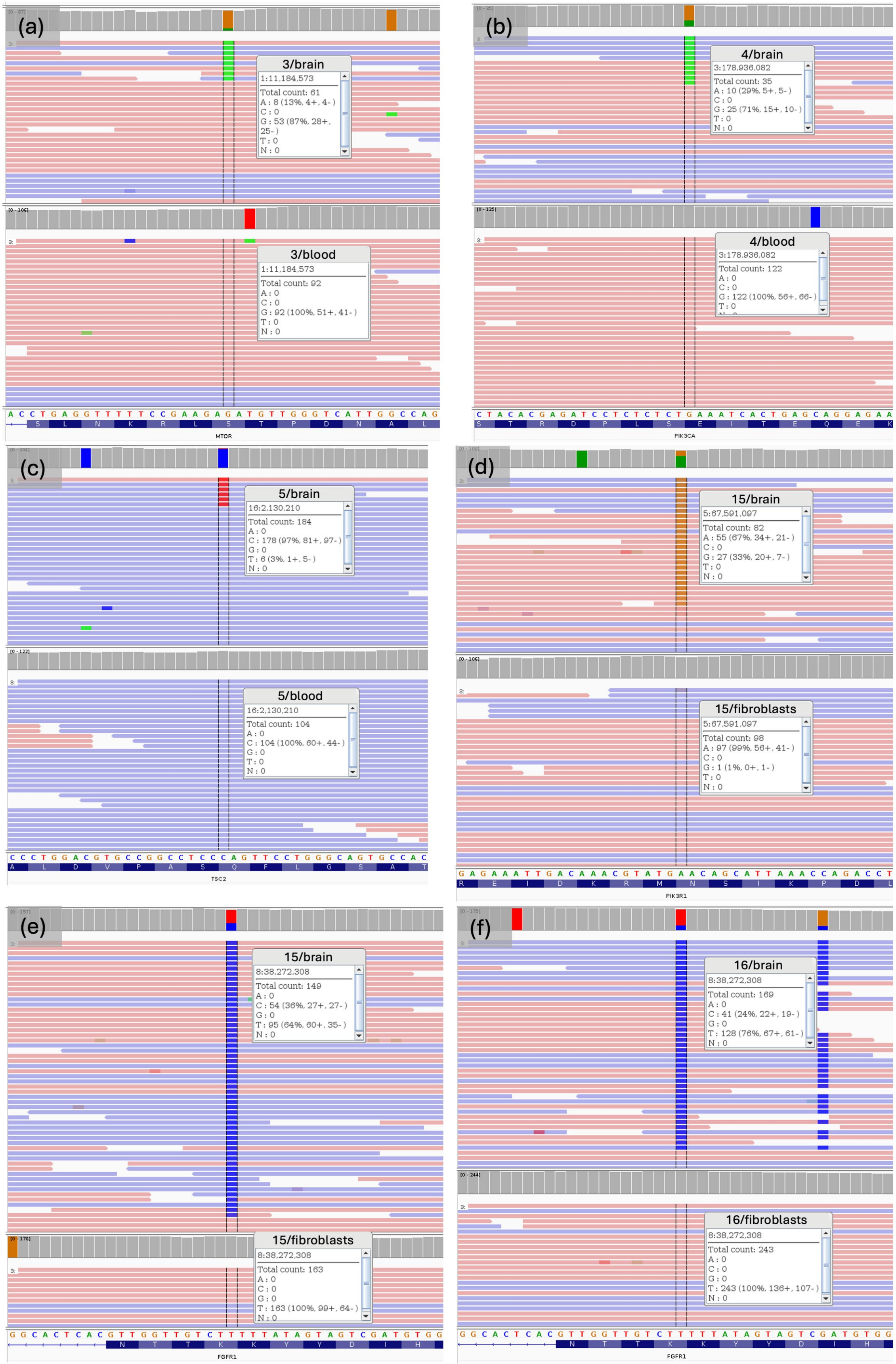

Figure S1. ***IGV screenshots of WES data with pathogenic somatic variants*** that were also confirmed with another, orthogonal method. The upper panel of each sub-image shows WES data on brain, the lower panel shows WES data on another reference tissue (blood or fibroblasts) The variant of interest is shown between the two vertical black dashed lines. **(a)** somatic variant in *MTOR* in individual 3 with HMEG, **(b)** somatic variant in *PIK3CA* in individual 4 with focal megalencephaly, **(c)** somatic variant in *MTOR* in individual 5 with FCDIIB, **(d)** and **(e)** somatic variants in *PIK3R1* and *FGFR1* in individual 15 with LGGNT, **(f)** somatic variant in *FGFR1* in individual 16 with DNET.

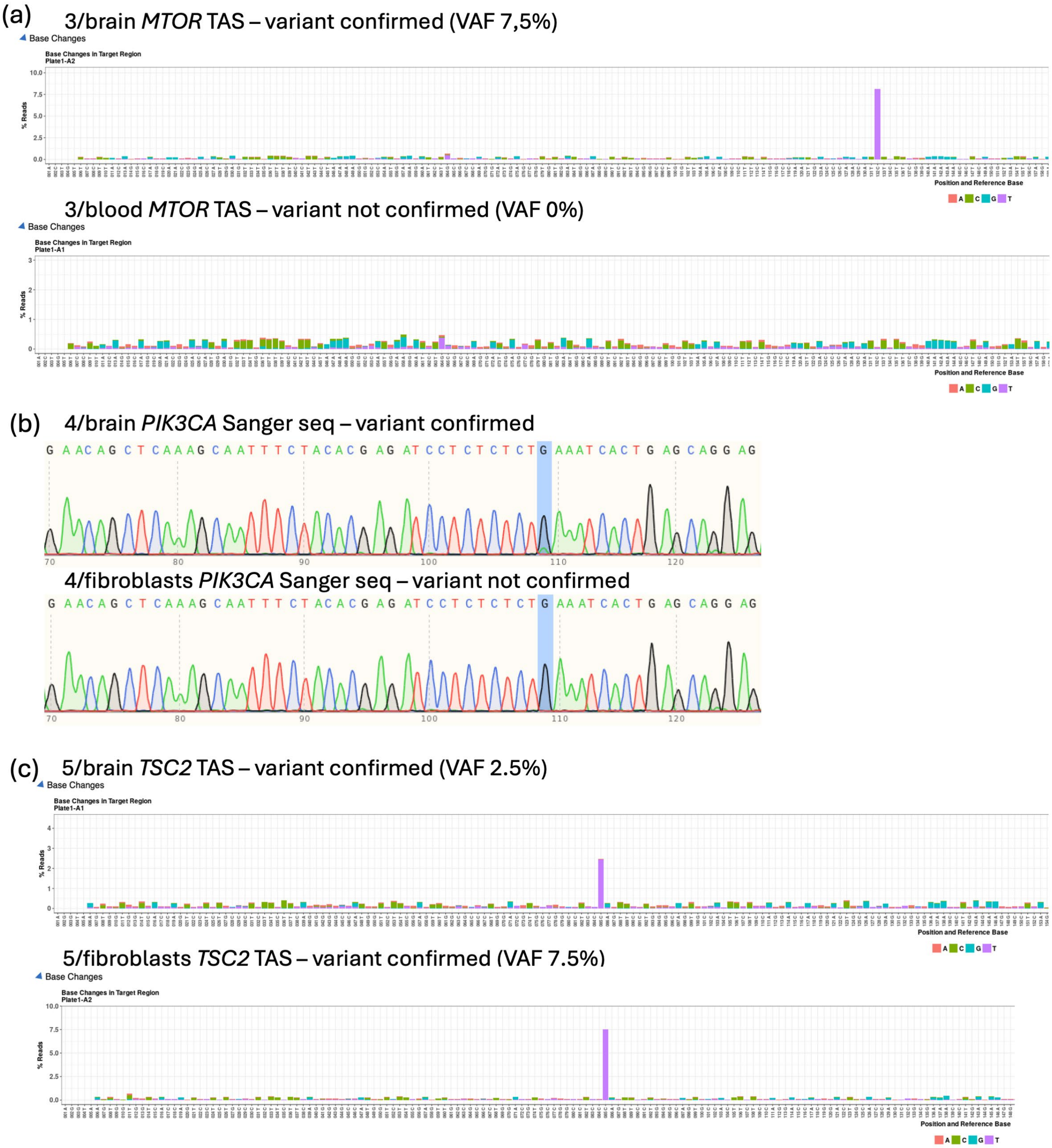

Figure S2/i. *Confirmation of detected somatic variants with an orthogonal method*. **(a)** Somatic variant in *MTOR* in individual 3, confirmed *via* TAS in brain with VAF 7.5%, not confirmed in blood, **(b)** somatic variant in *PIK3CA* in individual 4, confirmed *via* Sanger sequencing in brain, not confirmed in fibroblasts, no blood sample available for additional Sanger sequencing in blood, **(c)** somatic variant in *TSC2* in individual 5, confirmed *via* TAS in brain with VAF 2.5%, confirmed *via* TAS in fibroblasts with VAF 7.5%, no blood sample available for additional TAS in blood. Abbreviations: Sanger seq, Sanger sequencing; TAS, targeted amplicon sequencing; VAF, variant allele frequency.

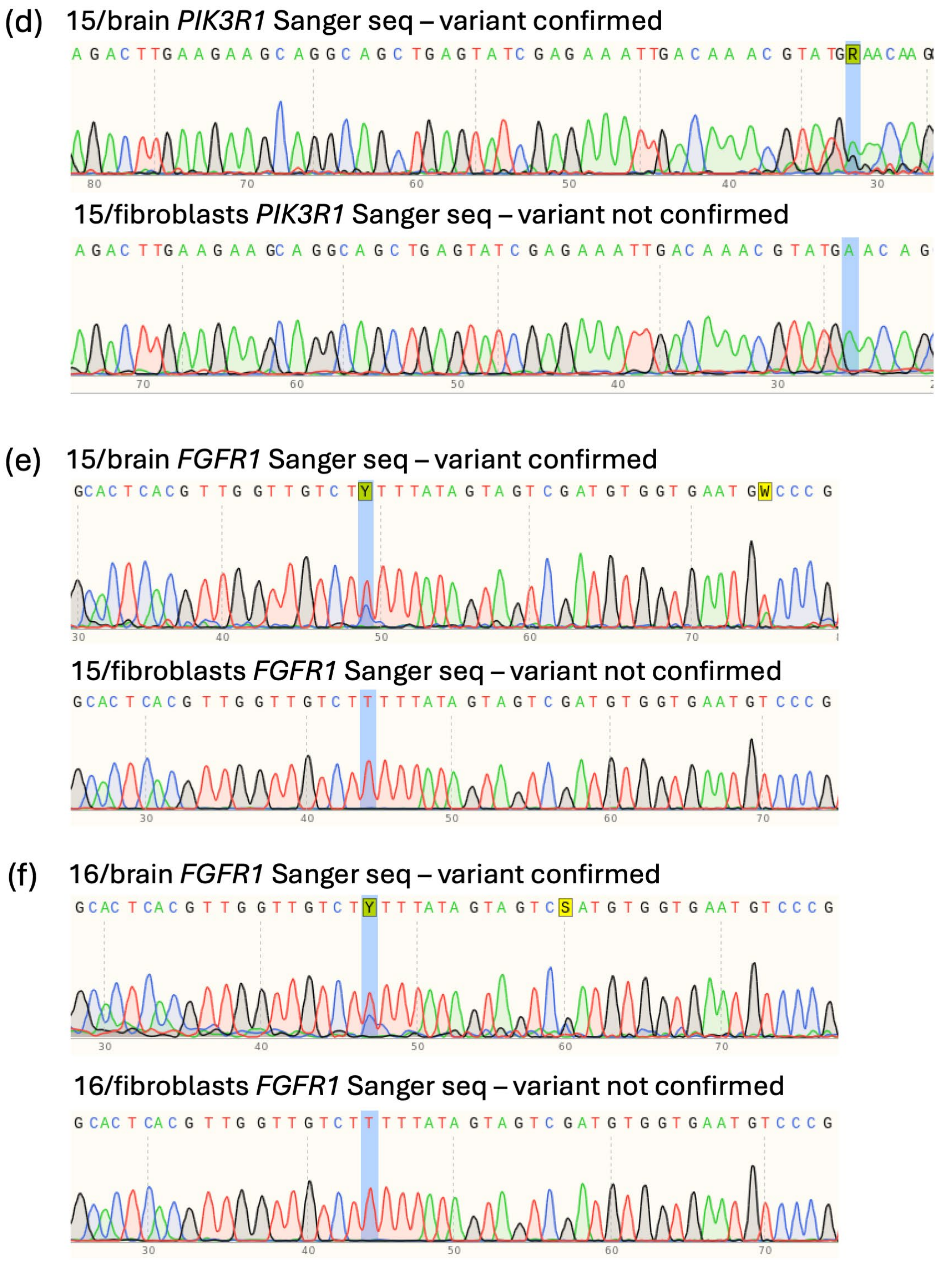

Figure S2/ii. *Confirmation of detected somatic variants with an orthogonal method.* **(d)** Somatic variant in *PIK3R1* in individual 15, confirmed *via* Sanger sequencing in brain, not confirmed in fibroblasts, no blood sample available for additional Sanger sequencing in blood, **(e)** somatic variant in *FGFR1* in individual 15, confirmed *via* Sanger sequencing in brain, not confirmed in fibroblasts, no blood sample available for additional Sanger sequencing in blood, **(f)** somatic variant in *FGFR1* in individual 16, confirmed *via* Sanger sequencing in brain, not confirmed in fibroblasts, no blood sample available for additional Sanger sequencing in blood. Abbreviations: Sanger seq, Sanger sequencing; TAS, targeted amplicon sequencing; VAF, variant allele frequency.

MTOR	Ct target-mu	Ct target-wt	Ct ref-gene	ratio wt/mu		
brain	26,94666667	21,02666667	18,57333333	0,016515907		> 1,6% VAF (percentage of mutated allele) in brain
blood	33,31666667	18,83333333	16,51666667	4,36598E-05		> 0% VAF (percentage of mutated allele) in blood
TSC2	Ct target-mu	Ct target-wt	Ct ref-gene	ratio wt/mu		
brain	26,66	20,81333333	20,58	0,017337023		>> 1,7% VAF (percentage of mutated allele) in brain
fibroblasts_2	24,22333333	20,23	19,63	0,06278948		>> 6,2% VAF (percentage of mutated allele) in fibroblasts_2

Figure S3. ***Results of DMAS-qPCR***. qPCR was run in triplicates, given C_t values are the mean of the three single C_t-values. Abbreviations: C_t target-mu = C_t values for gene of interest and allele-specific (AS) qPCR primers with mutation; C_t target-wt = C_t values for gene of interest and AS qPCR primers with wildtype; C_t ref-gene = C_t values for housekeeping/reference gene with respective primers; mu, mutated; wt, wildtype.
