# Supplementary material for: Somatic DNA Variants in Epilepsy Surgery Brain Samples from Patients with Lesional Epilepsy
Source: Int J Mol Sci. 2025 Jan 19;26(2):815. doi: 10.3390/ijms26020815 (PMC11766355; doi:10.3390/ijms26020815)
Supplement: Supplementary file 1 [file ijms-26-00815-s001.zip › 20241216_Supplementary_tables.pdf]

SUPPLEMENTARY TABLES to article

Somatic DNA Variants in Epilepsy Surgery Brain Samples from Patients with Lesional Epilepsy

Jana Marie Schwarz, Lena-Luise Becker, Monika Wahle, Jessica Faßbender, Ulrich W. Thomale, Anna Tietze, Susanne Morales-Gonzalez, Ellen Knierim, Markus Schuelke, Angela M. Kaindl

ACTB, ACTG1, ADGRG1, AKT1, AKT2, AKT3, AKT3, APC2, ARF1, ARFGEF2, ARX, ASPM, ATP1A2, ATP1A3, B3GALNT2, B4GAT1, CASK, CASP2, CCND2, CCND2, CDH2, CDK13, CDON, CEP85L, CNOT1, COL3A1, COL4A1, COL4A2, CRADD, CRPPA, CSNK2A1, CTNNA2, DAG1, DCHS1, DCX, DEPDC5, DHCR7, DISP1, DLL1, DPYSL5, DYNC1H1, EML1, EMX2, ENO1, EOMES, ERMARD, FAT4, FGF8, FGFR1, FIG4, FKRP, FKTN, FLNA, FOXH1, GCM2, GLI2, GMPPB, GPSM2, GRIN1, GRIN2B, H3-3A, HECTD4, HRAS, HS2ST1, KATNB1, KIF2A, KIF5C, KIFBP, KMT2D, KRAS, LAMA2, LAMB1, LAMC3, LARGE1, MACF1, MAP1B, MAPK8IP3, MAX, MN1, MTOR, MTOR, NDE1, NEDD4L, NODAL, NPRL2, NPRL3, NRAS, NSRP1, OCLN, OSGEP, PAFAH1B1, PEX1, PEX10, PEX11A, PEX11B, PEX12, PEX13, PEX14, PEX16, PEX19, PEX2, PEX26, PEX3, PEX5, PEX6, PEX7, PI4KA, PIDD1, PIK3CA, PIK3CA, PIK3R2, PIK3R2, PLCH1, POMGNT1, POMGNT2, POMK, POMT1, POMT2, PPP1R12A, PPP1R12A, PTCH1, PTEN, PTEN, RAB18, RAB3GAP1, RAB3GAP2, RAC3, RAD21, RELN, RTTN, RXYLT1, SCN3A, SHH, SIX3, SLC35A2, SMAD2, SMC1A, SMO, SNAP29, SOX11, STAG2, SUFU, TBC1D32, TBC1D7, TGIF1, TMX2, TP73, TSC1, TSC2, TUBA1A, TUBA8, TUBB, TUBB2A, TUBB2B, TUBB3, TUBG1, TUBGCP2, VLDLR, VPS50, WDR62, WNK3, ZIC2

Table S1: MCD gene panel

ACVR1, AKT1, APC, ARID1A, ATRX, BAP1, BCOR, BRAF, CDKN2A, CDKN2B, CIC, CTNNB1, CYSLTR2, DAXX, DDX3X, DGCR8, DICER1, DROSHA, EED, EGFR, EIF1AX, ERBB2, FGFR1, FGFR2, FGFR3, FUBP1, GNA11, GNAQ, GNAS, H3-3A, H3-3B, H3C14, H3C2, H3C3, HRAS, IDH1, IDH2, KBTBD4, KDM6A, KIT, KLF4, KRAS, LZTR1, MAP2K1, MET, MLH1, MSH2, MSH6, MTOR, NF1, NF2, NOTCH1, NRAS, NTRK1, NTRK2, NTRK3, PBRM1, PDGFRA, PIK3CA, PIK3R1, PLCB4, PMS2, POLD1, POLE, POLR2A, PPM1D, PRKAR1A, PRKCA, PTCH1, PTEN, PTPN11, RB1, SETD2, SF3B1, SMARCA2, SMARCA4, SMARCAL1, SMARCB1, SMARCE1, SMO, SUFU, SUZ12, TCF12, TERT, TP53, TRAF7, TSC1, TSC2, VHL

Table S2: Tumor gene panel

ABCC6, ACE, ACTA2, ACVRL1, ADA2, ADGRG1, ANGPTL6, ANIB1, ANTXR1, ARX, ATP7A, ATR, BRCC3, CBL, CCM2, CENPJ, CEP152, CEP63, CHD4, CNOT3, COL3A1, COL4A1, COL4A2, CRB1, CTSA, DCX, DNA2, ELN, ENG, EPHB4, FBN1, FLT4, FLVCR2, FOXF1, GDF2, GLA, GLMN, GNAQ, GUCY1A1, HBB, HLA-B, HLA-DQB1, HLA-DRB1, HTRA1, IL6, IRAG1, JAG1, KDR, KRIT1, LAMB1, LAMC3, LARGE1, MEF2C, MYH11, MYMY1, MYMY3, NDE1, NF1, NIN, NOTCH3, OCLN, OPHN1, PAFAH1B1, PCNT, PDCD10, PIK3CA, PIK3R2, PKD1, PKD2, POMGNT1, POMT1, POMT2, PTEN, RASA1, RBBP8, RELN, RNF213, RTTN, RXYLT1, SAMHD1, SETD5, SLC2A10, SMAD3, SMAD4, SMAD9, SMARCAL1, SRPX2, STAMBP, TEK, TGFB2, TGFBR1, TGFBR2, THSD1, TRAIP, TUBA1A, TUBA8, TUBB, TUBB2A, TUBB2B, TUBB3, TUBG1, VLDLR, WDR62, YY1AP1

Table S3: Vascular malformation gene panel

AARS1, AARS2, ABAT, ABCA2, ACOX1, ACTL6B, ADAM22, ADAR, ADARB1, ADAT3, ADD1, ADGRG1, ADGRL1, ADGRV1, ADPRS, ADRA2B, ADSL, AFF3, AFG2A, AFG2B, AGMO, AGO1, AIMP1, AIMP2, AJAP1, AKT1, AKT3, ALDH5A1, ALDH7A1, ALG1, ALG11, ALG12, ALG13, ALG14, ALG2, ALG3, ALG6, ALG8, ALG9, ALKBH8, ALPL, AMPD2, AMT, ANK2, ANKRD11, ANO4, AP1G1, AP2M1, AP3B2, APC2, ARF1, ARF3, ARFGEF1, ARFGEF2, ARG1, ARHGEF9, ARID1B, ARV1, ARX, ASAH1, ASH1L, ASL, ASNS, ASPA, ASTN1, ASXL3, ATN1, ATN1, ATP1A1, ATP1A2, ATP1A3, ATP2B1, ATP5F1A, ATP5PO, ATP6AP2, ATP6V0A1, ATP6V0A2, ATP6V0C, ATP6V1A, ATP7A, ATRX, BAP1, BCKDHA, BCKDHB, BCORL1, BCS1L, BET1, BLOC1S1, BLTP1, BOLA3, BORCS8, BRAF, BRAT1, BSCL2, BTD, C12orf57, C2orf69, CACNA1A, CACNA1B, CACNA1C, CACNA1D, CACNA1E, CACNA1G, CACNA1H, CACNA1I, CACNA2D1, CACNA2D2, CACNB4, CAD, CAMK2D, CAMK2G, CAMLG, CAMSAP1, CAPRIN1, CARS2, CASK, CASR, CBL, CC2D2A, CCDC186, CCDC88A, CCDC88C, CCND2, CDC42BPB, CDK19, CDKL5, CELF2, CEP85L, CERS1, CERT1, CHD2, CHD4, CHD5, CHKA, CHMP3, CHRM1, CHRNA2, CHRNA4, CHRNB2, CIC, CLCN2, CLCN3, CLCN4, CLCN6, CLDN5, CLN3,

CLN5, CLN6, CLN8, CLPB, CLTC, CNKSR2, CNNM2, CNOT9, CNPY3, CNTN2, CNTNAP2, COG3, COG4, COG6, COG7, COG8, COL18A1, COL4A1, COL4A2, COLGALT1, COQ2, COQ4, COQ6, COQ9, COX10, COX11, COX15, CPA6, CPLX1, CPSF3, CREBBP, CRELD1, CRH, CRPPA, CSNK1G1, CSNK2A1, CSNK2B, CSTB, CSTB, CTNNA2, CTSD, CTSF, CTU2, CUL3, CUL4B, CUX1, CUX2, CYFIP2, CYP27A1, D2HGDH, DALRD3, DBT, DCX, DDC, DDX3X, DEAF1, DEGS1, DENND5A, DENND5B, DEPDC5, DHCR24, DHCR7, DHDDS, DHPS, DHRSX, DHX16, DHX30, DIAPH1, DLL1, DMBX1, DMXL2, DNAJC5, DNAJC6, DNM1, DNM1L, DOCK7, DOLK, DPAGT1, DPH5, DPM1, DPM2, DPYD, DROSHA, DTYMK, DYNC1H1, DYRK1A, EARS2, EEF1A2, EFHC1, EFTUD2, EHMT1, EIF2A, EIF2AK2, EIF2B1, EIF2B2, EIF2B3, EIF2B4, EIF2B5, EIF2S3, EIF3F, EIF4A2, EMC10, EML1, EMX2, ENTPD1, EPG5, EPM2A, ESAM, ETHE1, EXOC7, EXOSC3, EXT2, FAM50A, FAR1, FARS2, FASTKD2, FBXL4, FBXO11, FBXO28, FCSK, FDFT1, FGF12, FGF13, FGFR3, FH, FIG4, FKRP, FKTN, FLNA, FOLR1, FOXG1, FOXRED1, FRMD5, FRRS1L, FTL, FUCA1, FUT8, FZR1, GABBR2, GABRA1, GABRA2, GABRA5, GABRB1, GABRB2, GABRB3, GABRD, GABRG2, GAD1, GAL, GALC, GALNT2, GAMT, GATAD2B, GATM, GBA1, GCH1, GCSH, GFAP, GFM1, GLB1, GLDC, GLI3, GLRA1, GLRA2, GLRB, GLS, GLUD1, GLUL, GLYCTK, GM2A, GNAO1, GNAQ, GNB1, GNB2, GNB5, GOSR2, GOT2, GPAA1, GPHN, GRIA2, GRIA4, GRIK2, GRIN1, GRIN2A, GRIN2B, GRIN2D, GRM7, GRN, GSS, GTPBP2, GTPBP3, GUF1, H3-3A, H3-3B, HACE1, HAX1, HCCS, HCFC1, HCN1, HCN2, HEATR5B, HECTD4, HECW2, HEPACAM, HERC2, HEXA, HEXB, HID1, HLCS, HMGCL, HNRNPH2, HNRNPR, HNRNPU, HOXA1, HPDL, HPRT1, HRAS, HSD17B10, HSD17B4, HSPD1, HTRA2, IDH2, IER3IP1, IFIH1, IKBKG, INO80, IQSEC2, IRF2BPL, ITPA, JAKMIP1, KARS1, KAT5, KAT8, KATNB1, KCNA1, KCNA2, KCNA3, KCNB1, KCNB2, KCNC1, KCNC2, KCND2, KCNH1, KCNH5, KCNJ10, KCNJ11, KCNK4, KCNMA1, KCNQ2, KCNQ3, KCNQ5, KCNT1, KCNT2, KCTD3, KCTD7, KDM6B, KIF1A, KIF2A, KIF5C, KIFBP, KLHL20, KMT2E, KMT5B, KPTN, KRAS, LARGE1, LARS1, LETM1, LGI1, LIAS, LIPT1, LIPT2, LMAN2L, LMBRD2, LMNB1, LMNB2, LNPK, LSS, LYST, MACF1, MADD, MAF, MAGI2, MANBA, MAP2K1, MAP2K2, MAPK10, MAST1, MAST3, MAST4, MATN4, MBD5, MBOAT7, MCM3AP, MDH2, MECP2, MED11, MED12, MED17, MED27, MEF2C, MFF, MFSD8, MINPP1, MLC1, MMACHC, MMADHC, MOCS1, MOCS2, MOGS, MPDU1, MT-CO3, MT-TL1, MTHFR, MTHFS, MTOR, MTR, MYO1H, NACC1, NAGA, NAPB, NARS1, NARS2, NBEA, NCDN, NDE1, NDP, NDUFA1, NDUFA10, NDUFA11, NDUFA2, NDUFAF2, NDUFAF3, NDUFAF4, NDUFAF5, NDUFS1, NDUFS2, NDUFS4, NDUFS6, NDUFS7, NDUFS8, NDUFV1, NECAP1, NEDD4L, NEUROD2, NEXMIF, NGLY1, NHLRC1, NID1, NPRL2, NPRL3, NR4A2, NRAS, NRROS, NRXN1, NSD1, NSDHL, NSF, NSRP1, NTRK2, NUBPL, NUP214, NUS1, OCLN, OGDHL, OPHN1, OTUD6B, OTUD7A, OTX2, OXR1, P4HTM, PABPC1, PACS1, PACS2, PAFAH1B1, PAH, PAK1, PARP6, PARS2, PCCA, PCCB, PCDH12, PCDH19, PCDHB4, PCDHGC4, PCLO, PCYT2, PDHA1, PDHX, PDSS2, PET100, PEX1, PEX10, PEX12, PEX13, PEX19, PEX2, PEX3, PEX5, PEX6, PEX7, PGM2L1, PHACTR1, PHGDH, PIDD1, PIGA, PIGB, PIGC, PIGG, PIGH, PIGK, PIGM, PIGN, PIGO, PIGP, PIGQ, PIGS, PIGT, PIGU, PIGW, PIK3CA, PIK3R2, PIP5K1C, PLA2G6, PLAA, PLCB1, PLK1, PLPBP, PLPBP, PLXNA1, PMM2, PMPCB, PNKP, PNPO, PNPT1, POLG, POLG2, POMGNT1, POMT1, POMT2, PPFIBP1, PPIL1, PPP1R3F, PPP2CA, PPP3CA, PPT1, PRDM8, PRICKLE1, PRICKLE2, PRMT7, PRODH, PRPF8, PRRT2, PSAP, PSAT1, PSMB8, PSPH, PTCD3, PTCH1, PTEN, PTF1A, PTPN23, PTS, PUM1, PURA, QARS1, QDPR, RAB11A, RAB11B, RAB18, RAB3GAP1, RAB3GAP2, RAB5C, RAC3, RALA, RALGAPA1, RALGAPB, RANBP2, RARS1, RARS2, RELN, RFT1, RHEB, RHOBTB2, RMND1, RNASEH2A, RNASEH2B, RNASEH2C, RNASET2, RNF113A, RNF13, RNF2, RNU2-2P, RNU4-2, RNU4ATAC, ROGDI, RORA, RORB, RPIA, RRM2B, RTN4IP1, RTTN, RUBCN, RUSC2, RYR2, RYR3, SAMD12, SAMHD1, SARS1, SATB1, SATB2, SCAF4, SCAMP5, SCARB2, SCN1A, SCN1B, SCN2A, SCN2B, SCN3A, SCN8A, SCN9A, SCO1, SCO2, SDHA, SEC24D, SEC31A, SEMA6B, SEPSECS, SERPINI1, SETBP1, SETD1A, SETD1B, SETD5, SGSH, SHH, SHQ1, SIK1, SIX3, SLC12A5, SLC13A5, SLC16A2, SLC1A2, SLC1A4, SLC25A1, SLC25A12, SLC25A19, SLC25A22, SLC2A1, SLC31A1, SLC32A1, SLC35A1, SLC35A2, SLC35A3, SLC38A3, SLC39A8, SLC45A1, SLC5A6, SLC6A1, SLC6A19, SLC6A5, SLC6A8, SLC7A6OS, SLC9A6, SMARCA2, SMARCC2, SMC1A, SMS, SNAP25, SNF8, SNIP1, SNORD118, SNX27, SPR, SPTAN1, SPTBN1, SPTBN4, SRPX2, ST3GAL3, ST3GAL5, STAG1, STAMBP, STARD7, STIL, STRADA, STX1B, STXBP1, SUCLA2, SUCLG1, SUOX, SURF1, SYN1, SYNCRIP, SYNGAP1, SYNJ1, SZT2, TAF8, TANC2, TANGO2, TBC1D20, TBC1D24, TBC1D2B, TBCD, TBCK, TBL1XR1, TCF4, TDP2, TEFM, TELO2, TET3, TFE3, TGIF1, TIAM1, TIMM50, TMEM106B, TMEM222, TMEM63B, TMEM70, TMX2, TNK2, TNPO2, TPP1, TRA2B, TRAF7, TRAK1, TRAPPC12, TRAPPC4, TRAPPC6B, TREX1, TRIM8, TRIP13, TRIT1, TRPM3, TRPM6, TRRAP, TSC1, TSC2, TSEN15, TSEN2, TSEN34, TSEN54, TSFM, TUBA1A, TUBA3E, TUBA8, TUBB, TUBB2A, TUBB2B, TUBB3, TUBB4A, TUBG1, TUBGCP2, TXN2, TXNRD1, U2AF2, UBA5, UBAP2L, UBE2A, UBE3A, UBR7, UFC1, UFM1, UFSP2, UGDH, UGP2, UNC13B, UNC80, USP18, USP7, VAMP2, VARS1, VLDLR, VPS11, VPS50, WARS2, WASF1, WDR37, WDR45, WDR45B, WDR62, WDR73, WNK3, WWOX, XK, YIF1B, YIPF5, YWHAG, ZBTB18, ZBTB47, ZDHHC9, ZEB2, ZIC2, ZMIZ1, ZMYM2, ZNF142, ZNF335, ZNFX1

Table S4: Epilepsy gene panel

ACTA2, ADA, ADA2, BMPR2, CARD14, CBL, CBS, COL3A1, COL4A1, ELN, FBN1, FBN2, FOXE3, FOXP3, GP6, GUCY1A1, HTRA1, LOX, MAT2A, MFAP5, MYH11, MYLK, NOTCH1, NOTCH3, PRKG1, RNF213, SAMD3, SKI, SLC2A10, SMAD2, SMAD3, SMAD4, TGFB2, TGFB3, TGFB1, TGFBR1, TGFBR2, USB1, YY1AP1, COPA, CTLA4, IL1RN, LPIN2, MEFV, MVK, NLRP12, NLRP3, NOD2, OTULIN, PLCG2, PLOD1, PSMA3, PSTPIP1, PYCARD, RBCK1, TNFAIP3,

TNFRSF1A, HSP90B3P, TRIM28, TRNT1, WDR1, C1QA, C1QB, C1QC, C1R, C2, C2, C3, C5, C6, C7, C8A, C8B, CFH, CFHR5, CFI, CTPS1, MASP2, RANBP2, NLRC4, PRF1, SH2D1A, SLC29A3, STX11, STXBP2, UNC13D, XIAP, CLEC16A, FAS, GATA2, IRF5, IRF8, ITPR3, MYO5A, PTPN22, RHOD, STAT3, STAT4, TBK1, ACP5, ADAR, RIGI, DNASE1, DNASE1L3, DNASE2, IFIH1, ISG15, NDUFS1, NDUFS2, NDUFS3, NDUFS4, NDUFS5, NDUFS6, NDUFS7, NDUFS8, POMP, PRKCD, PSMB3, PSMB4, PSMB8, PSMB9, RAB27A, RNASEH2A, RNASEH2B, RNASEH2C, SAMHD1, SKIC2, STAT2, STING1, TREX1, USP18, EGR2, LMNA, LMNB1, GJB1, MFN2, MPZ, NEFL, NF1, PMP22, PRX, TSC1, TSC2, SKIC3, LYST, ATP7B, CORO1A, CPT2, FOLR1, HMBS, PHYH, ARX, ISCA2, AARS2, ABCD1, ACOX1, COQ3, AIFM1, AIMP1, ALDH3A2, ARSA, ASPA, ATPAF2, BCS1L, BOLA3, BTD, CLCN2, COQ2, COQ9, COX10, COX15, CSF1R, CST3, CTC1, CYP27A1, DARS1, DARS2, DGUOK, EARS2, EIF2B1, EIF2B2, EIF2B3, EIF2B4, ERCC2, ERCC3, ERCC6, ERCC8, ETFDH, EXOSC8, HYCC1, FUCA1, FXN, GALC, GBE1, GFAP, GFM1, GJC2, GLA, GTF2H5, HEPACAM, HFE, HSD17B4, IBA57, LONP1, MPLKIP, MRPS16, NDUFAF1, NDUFV1, NUBPL, PARN, PDHA1, PEX1, PEX10, PEX11A, PEX11B, PEX12, PEX13, PEX14, PEX16, PEX3, PEX2, PEX26, PEX5, PEX7, PEX6, PGM3, PLP1, POLG, POLG2, POLR1C, POLR3A, POLR3B, PSAP, PYCR2, RARS1, RASGRP1, RNASET2, RRM2B, SCN9A, SCO1, SCO2, SCP2, SDHAF1, SDHB, SLC16A2, SLC17A5, SLC1A4, SLC25A12, SOX10, SPTAN1, SUCLA2, SUMF1, SURF1, TACO1, TUBB4A, TUFM, TYMP, NFU1, SNORD118

Table S5: Autoinflammation gene panel

ABCA1, ABCA11P, ABCA2, ABCA3, ABCA4, ABCA5, ABCA6, ABCA7, ABCA8, ABCB1, ABCB10, ABCB11, ABCB4, ABCB5, ABCB6, ABCB7, ABCB8, ABCB9, ABCC1, ABCC10, ABCC11, ABCC12, ABCC13, ABCC2, ABCC3, ABCC4, ABCC5, ABCC6, ABCC8, ABCC9, ABCD1, ABCD2, ABCD3, ABCD4, ABCE1, ABCF1, ABCF2, ABCF3, ABCG1, ABCG2, ABCG4, ABCG5, ABCG8, AOC1, ADH1A, ADH1B, ADH1C, ADH4, ADH5, ADH6, ADH7, ADHFE1, AHR, AHRR, AKR1C1, AKR1C2, AKR1C3, AKR1C4, ALDH1A1, ALDH1A2, ALDH1A3, ALDH1B1, ALDH2, ALDH3A1, ALDH3A2, ALDH3B1, ALDH3B2, ALDH4A1, ALDH5A1, ALDH6A1, ALDH7A1, ALDH8A1, ALDH9A1, AOX1, ARNT, ARSA, ATP7A, ATP7B, BSG, CABIN1, CARM1, CAT, CAV1, CBR1, CBR3, CDA, CEBPA, CES1, CES2, CFTR, CHST1, CHST10, CHST11, CHST12, CHST13, CHST2, CHST3, CHST4, CHST5, CHST6, CHST7, CHST8, CHST9, CHURC1, COMT, CREBBP, CRP, CYB5R3, CYP11A1, CYP11B1, CYP11B2, CYP17A1, CYP19A1, CYP1A1, CYP1A2, CYP1B1, CYP20A1, CYP21A2, CYP24A1, CYP26A1, CYP26C1, CYP27A1, CYP27B1, CYP2A13, CYP2A6, CYP2A7, CYP2B6, CYP2B7P, CYP2C18, CYP2C19, CYP2C8, CYP2C9, CYP2D6, CYP2D7, CYP2E1, CYP2F1, CYP2J2, CYP2R1, CYP2S1, CYP39A1, CYP3A4, CYP3A43, CYP3A5, CYP3A7, CYP46A1, CYP4A11, CYP4B1, CYP4F11, CYP4F12, CYP4F2, CYP4F3, CYP4F8, CYP4Z1, CYP51A1, CYP7A1, CYP7B1, CYP8B1, DDO, DHRS1, DHRS12, DHRS13, DHRS2, DHRS3, DHRS4, DHRS4L1, DHRS4L2, DHRS7, DHRS7B, DHRS7C, DHRS9, DHRSX, DPEP1, DPYD, EAF2, EP300, EPHX1, EPHX2, EPS8L3, ESR1, ESR2, ESRRA, ESRRB, ESRRG, FMO1, FMO2, FMO3, FMO4, FMO5, FMO6P, FOXA2, FOXA3, GPS2, GPX1, GPX2, GPX3, GPX4, GPX5, GPX6, GPX7, GSR, GSS, GSTA1, GSTA2, GSTA3, GSTA4, GSTA5, GSTCD, GSTK1, GSTM1, GSTM2, GSTM3, GSTM4, GSTM5, GSTO1, GSTO2, GSTP1, GSTT1, GSTT2, GSTZ1, HAGH, HIF1A, HNF1A, HNF4A, HNMT, HSD11B1, HSD17B11, HSD17B14, IAPP, IL6ST, INSIG1, INSIG2, KCNJ11, LOC728667, LOC731356, MAT1A, METAP1, MGMT, MGST1, MGST2, MGST3, MIF, MPO, NAT1, NAT2, NCOA1, NCOA2, NCOA3, NCOA6, NCOR1, NCOR2, NNMT, NOS1, NOS2, NOS3, NR0B2, NR1H2, NR1H3, NR1H4, NR1I2, NR1I3, NR2C1, NR2C2, NR2F1, NR2F2, NR3C1, NR3C2, NR5A2, NUDT8, PDE3A, PDE3B, PGRMC1, PGRMC2, PIAS2, PLG, PLGLB1, PNMT, PON1, PON2, PON3, POR, PPARA, MED1, PPARD, PPARG, PPARGC1A, PRMT1, RARα, RNF40, RXRA, SERPINA7, SIRT1, SLC10A1, SLC10A2, SLC13A1, SLC13A2, SLC13A3, SLC15A1, SLC15A2, SLC16A1, SLC16A3, SLC16A7, SLC19A1, SLC22A1, SLC22A10, SLC22A11, SLC22A12, SLC22A13, SLC22A14, SLC22A15, SLC22A16, SLC22A17, SLC22A18, SLC22A18AS, SLC22A2, SLC22A25, SLC22A3, SLC22A4, SLC22A5, SLC22A6, SLC22A7, SLC22A8, SLC22A9, SLC27A1, SLC28A1, SLC28A2, SLC28A3, SLC29A1, SLC29A2, SLC29A4, SLC2A4, SLC2A5, SLC47A1, SLC47A2, SLC5A12, SLC5A6, SLC6A6, SLC7A5, SLC7A7, SLC7A8, SLCO1A2, SLCO1B1, SLCO1B3, SLCO1C1, SLCO2A1, SLCO2B1, SLCO3A1, SLCO4A1, SLCO4C1, SLCO5A1, SLCO6A1, SOD1, SOD2, SOD3, SQSTM1, STAT3, STK19, SULF1, SULT1A1, SULT1A2, SULT1A3, SULT1B1, SULT1C2, SULT1E1, SULT2A1, SULT2B1, SULT4A1, TAP1, TAP2, TPMT, TRPC1, TRPC3, TRPC4, TRPC5, TRPC6, TRPC7, TRPM1, TRPM6, TRPM7, TRPV4, TYMS, UGT1A1, UGT1A10, UGT1A3, UGT1A4, UGT1A5, UGT1A6, UGT1A7, UGT1A8, UGT1A9, UGT2A1, UGT2B10, UGT2B11, UGT2B15, UGT2B17, UGT2B28, UGT2B4, UGT2B7, UGT8, UROC1, VDR, VKORC1, XDH, XRCC5

Table S6: ADME gene panel

Gene	FOR oligonucleotide primer	REV oligonucleotide primer
FGFR1	TGGTGACAGAGGACAATGTGA	CCACCCCACTCCTTGCTTC
PIK3CA	ATCTGGTCTTGTTGTTGGCTA	CTGAGATCAGCCAAATTCAGT
PIK3R1	AGTTGAAGTCTCGAATCAGTGA	AGTATTGGTCTCTCGTCTTTCTC

Table S7: Sanger sequencing primers. FOR, forward; REV, reverse

Gene	REV allele-specific mu primer	REV allele-specific wt primer	common FOR primer
MTOR	CTGAGGTTTTTCCGACGAA	CTGAGGTTTTTCCGACGAG	CAGCAACGGACATGAGTTTG
TSC2	TGGCACTGCCCAGGATCTA	TGGCACTGCCCAGGATCTG	GAGGCTGGTGGTTTTGCATC
	**common FOR primer**	**common REV primer**	
GAPDH	CTGGTAAAGTGGATATTGTTGCCAT	TGGAATCATATTGGAACATGTAAACC	

Table S8: DMAS qPCR primers. FOR, forward; mu, mutant; REV, reverse; wt, wildtype

Gene	FW oligonucleotide primer	REV oligonucleotide primer
DAB1	TTCTTTCCCAGGACACCAGG	GCTTGTCGGTCTGTGGACTT
METAP1	TTTCCTTGGCTTTCTTCTTGATAG	GTCGCCGGGTTAGGATTTCA
TRPV4	TGATGGTCTTTGCCCTGGTC	GTGTGTGTGACTCCCTCCAG
TSC2	GAGGCTGGTGGTTTTGCATC	GTGGGTGACTGGCAGAAAGA
MTOR	GATGTACACTCACCGCTCCC	CTGCCTCAACCTCTGGAGTT
PAK6	GGGACAAGGGAACAGGAAGT	CAGGCATACTTGGGGTGGG
NF1	TGTCTTCAGAGGGAAACGCA	CTAAGAGGCAAGCTGACCCC
APC2	TCCAAACCTAACCAGATGCGT	CCATGGCGATCATCTTGTGC

Table S9: TAS primers, FOR, forward; REV, reverse

Brain sample/ individual	Group	Gene / variant	VAF brain WES	VAF blood / fibroblast WES	Validation method / VAF brain
10	**B**	*TRPV4* c.1796C>T, p.T599M, NM_021625	0.7%	blood 0%	TAS 0%
11	**B**	*APC2* c.1940G>A, p.G647D , ENST00000233607.2	1.0%	blood 0%	TAS 0%
13	**C**	*DAB1* c.1054C>T, p.Q352*, NM_001365792	2.7%	blood 0%	TAS 0%
14	**D**	*PAK6* c.1916T>A, p.L639Q, ENST00000560346	1.0%	blood 0%	TAS 0%
16	**E**	*NF1* c.2824delA, p.S942Afs*, NM_001042492	4.5%	fibroblast 0%	TAS 0%
18	**E**	*METAP1* c.1037C>T, p.A346V, NM_015143	0.8%	blood 0%	TAS 0%

Table S10: Unconfirmed variants. VAF, variant frequency
